# Are There Two Kinds of Reasoners?

**DOI:** 10.3390/jintelligence12030025

**Published:** 2024-02-22

**Authors:** Henry Markovits

**Affiliations:** Département de Psychologie, Université du Québec à Montréal, Montréal, QC H3C 3P8, Canada; markovits.henry@uqam.ca

**Keywords:** reasoning, dual strategy, individual differences, mental models, probabilistic theories

## Abstract

There is little consensus about the underlying parameters of human reasoning. Two major theories have been proposed that suppose very different mechanisms. The mental model theory proposes that people use working memory intensive processes in order to construct limited models of problem parameters. Probabilistic theories propose that reasoning is a process by which people use the sum of their existing knowledge in order to generate an estimate of the probability of a conclusion given problem parameters. Following an initial proposition by Verschueren et al., the dual-strategy model supposes that these different approaches to reasoning are in fact an important individual difference. Specifically, a recently developed diagnostic questionnaire has identified two major categories of reasoners: **Counterexample** reasoners use a mental model form of processing, while **Statistical** reasoners use a probabilistic form of processing. In the following, I describe results that show that the Counterexample/Statistical distinction affects information processing across a variety of reasoning and judgment tasks. In addition, strategy use correlates with performance on very different kinds of thinking, such as contingency judgments, processing of negative emotions, or susceptibility to social biases. Although this distinction is related to differences in cognitive ability, it has been found to predict performance over and above these differences. More recent results have shown that it is possible to experimentally modify strategy use. These results suggest that strategy use is an important individual difference that can affect performance in a wide variety of contexts.

## 1. Are There Two Kinds of Reasoners?

Deductive reasoning in its most simple form involves generating a conclusion based on a given set of premises. Such reasoning is both a core element of the development of advanced mathematical and scientific models and is a ubiquitous component of ordinary social interactions. Understanding the nature of human reasoning is thus one of the core dimensions of any model of human intelligence. There is, however, no real consensus about the underlying processes that define how people generate conclusions. In the following, I present evidence that there is a qualitative distinction between two different modes of reasoning that form a strong individual difference, one that is instantiated in the dual-strategy model of reasoning.

Underlying this model is a debate between two major theories of reasoning, mental model, and probabilistic theories. The mental model theory (Hinterecker et al. 2016; Johnson-Laird 2001; Johnson-Laird 2012) postulates that reasoning involves the active construction of simple, iconic models of problem premises. Although the full theory is much more complex than will be presented here, three critical components are of particular concern here. The first is that models are simplified representations that focus on key aspects of a given deductive problem. The second is that such models are actively generated in working memory and require significant cognitive resources. The final aspect is that when attempting to generate a putative conclusion from a given model set, people will examine this for any potential counterexamples: a conclusion is considered to be valid if and only if the model set does not contain any cases that contradict this conclusion. The mental model theory is based on the underlying idea that the semantics of logical connectors (if-then, etc.) generally correspond to the truth-table interpretation of these connectors in standard logics. Deviations from standard logic are explained by cognitive capacity, since manipulating models requires significant short term memory usage, and by other factors such as pragmatic considerations (Johnson-Laird 2001) which may modulate the nature of the models that are generated.

The mental model theory, despite the existence of factors that could in principle explain the very great amount of variability observed when people reason is empirically examined, remains tied to the structures of propositional logic. Probabilistic theories, on the other hand, attempt to incorporate variability directly into the underlying structure of reasoning. Although there are different varieties, the basic model is that when faced with some premises, people will derive an intuitive probability of some putative conclusion (Evans et al. 2003; Oaksford and Chater 2009; Oaksford et al. 2000). This will be performed not only with respect to the premises, but also by considering what people know about the premises. These theories attempt to model the nature of informal reasoning, which very often is expressed as when “something is more or less probable” to some degree. Underlying these theories is an essentially Bayesian model of probabilistic updating. These suggest that when evaluating a potential conclusion, people make intuitive evaluations that incorporate their understanding of how the world works. Deduction is then an inherently probabilistic enterprise. Dichotomous judgments of validity are, in this perspective, not a natural outcome of this kind of reasoning. When making a deduction, the intuitive probability that is assigned to a putative conclusion must be translated into a judgment of validity, but one that maintains the basic information generated by the initial probabilistic evaluation.

These theories have been proposed as unitary models of human reasoning, each of which has claimed to be able to understand, with some adjustments, how people reason. However, Verschueren and colleagues suggested that these divergent theories could be seen as different ways of reasoning (Verschueren et al. 2005a, 2005b). They incorporated this hypothesis within the context of dual-process theories which postulate the existence of two different reasoning systems (Evans and Stanovich 2013; Evans 2007; Sloman 1996; Stanovich and West 2000). Briefly, Type 1 reasoning is essentially rapid, intuitive, and associative in nature, while Type 2 reasoning is more conscious and working memory intensive. Within this context, they proposed that probabilistic reasoning could be seen as a form of Type 1 processing, while mental model-like reasoning was a form of Type 2 processing. In other words, they proposed that people can make both a probabilistic judgment that considers the statistical distribution of potential alternative antecedents in order to generate an estimate of the likelihood of a potential conclusion being true, and categorical judgments of validity for which a single potential counterexample is sufficient to reject a potential conclusion. They examined verbal protocols and found evidence for use of both counterexamples and likelihood estimates. In addition, there was evidence that use of a probabilistic strategy was relatively greater among reasoners with lower working memory spans, i.e., that less competent reasoners will tend to produce a likelihood estimate more often. Finally, they found that access to counterexample information was a slower process than generating likelihood estimates. Their results provide a useful basis for the proposed distinction. However, the use of verbal protocols might have an influence on the way that people reason and does not allow for a clear way of distinguishing the two ways of reasoning.

Markovits et al. (2012) proposed a more direct way of distinguishing these two ways of reasoning. This involves presenting participants with simple conditional reasoning problems using an affirmation of the consequent inference (P implies Q, Q is true), for which there is no valid logical response. Reasoners were asked if the putative conclusion “P is true” was valid or not. In order to reduce the extent to which existing knowledge might impact inferences, premises referred to unknown elements situated on a different planet. The key addition was the results of 1000 observations made on the planet that concerned the if-then relation used in the major premise, based on a previous study by (Geiger and Oberauer 2007). This gave the number of times that P and Q were observed together, and the number of times that Q was observed but not P. Critically, this gives two kinds of information. The ratio of (P and Q) to the total gives the statistical probability of the putative conclusion being true. The existence of a non-zero number of (not-P and Q) cases indicates the presence of counterexamples to this conclusion. Now, since there is no clear algorithm for translating a probabilistic evaluation into a dichotomous validity judgment, presenting a single inference of this kind would not be diagnostic. The key was presenting two sets of five such inferences, one of which presented about a 90% rate of (P and Q) cases, with the other presenting about a 50% rate of these cases. A reasoner who used the statistical information would end up accepting the 90% inferences more often than the 50% inferences, at a rate that would generally reflect the difference in probabilities. A reasoner who focused on potential counterexamples would reject all of these inferences. This diagnostic instrument has been used to distinguish between reasoners who use the statistical information provided to make inferences (who are referred to as Statistical reasoners) and those who use the provided information as indicators of the existence of potential counterexamples (who are referred to as Counterexample reasoners), see Appendix A for full details.

This diagnostic instrument is the basis for the dual-strategy model of reasoning, which supposes that people have a preferred mode of reasoning corresponding to either a Statistical or a Counterexample strategy. Initial studies concentrated on validation of this basic idea. The results of (Markovits et al. 2012) provided clear initial support for the usefulness of the diagnostic instrument, showing that reasoners identified as using a Counterexample strategy showed better abstract reasoning abilities than those identified as using a Statistical strategy. More direct were the results of (Markovits et al. 2013), which examined performance on the diagnostic instrument under both unlimited and time constrained conditions. In addition, participants were asked for their level of confidence in their overall choices. The key results showed that under time constraint, people preferentially responded to the diagnostic instrument with responses that indicated used of a Statistical strategy, even when they responded with a Counterexample pattern when given unlimited time. In addition, transitions under different time conditions were predicted by people’s confidence in their initial strategy choice. These results provided strong evidence for the idea that people have access to different ways of reasoning, and that these were under some degree of metacognitive control.

Subsequent studies have shown that strategy use as defined by the diagnostic instrument is correlated with a great variety of forms of reasoning and judgment, in a way that strongly suggests that this is a basic individual difference. Before examining these results, it was necessary to investigate an alternative hypothesis for performance on the diagnostic questionnaire. In this, reasoners are categorized as using a Counterexample strategy if they reject all of the AC inferences. The underlying model suggests that they are doing so because they are sensitive to the importance of potential counterexamples to the presented inferences. However, since according to standard propositional logic, there is no single valid conclusion to any AC inference; another possible explanation could be that Counterexample reasoners are simply responding to the logical form of the inference. In other words, the diagnostic instrument might simply be distinguishing people who use formal logic from those who do not. In order to examine this alternative explanation, Markovits et al. (2016) presented reasoners with Modus ponens inferences (P implies Q, P is true, Is Q true?) for which the conclusion “Q is true” is logically valid. However, when paired with potential counterexamples, reasoners categorized as using a Counterexample strategy were less “logical” than Statistical reasoners; i.e., they endorsed the logical conclusion less often. In other words, it is not possible to attribute the responses given by Counterexample reasoners as being due to a greater ability to respond according to logical form. This is a key result, since it suggests that the distinction between Counterexample and Statistical strategies is the result of different ways of processing information, and not simply due to the former being more attuned to the parameters of formal logic.

## 2. Strategy and Cognitive Capacity

A second, more complex but related question is the relation between cognitive capacity and reasoning strategy. The initial studies by (Verschueren et al. 2005b) suggested that people whose explicit justifications indicated use of a mental model-like form of reasoning had higher levels of working memory capacity. Later studies have indeed shown a strong correlation between several measures of cognitive capacity and strategy use. In (Thompson and Markovits 2021), the authors observed that Counterexample reasoners had higher scores on measures of IQ and the Cognitive Reflection Test (CRT). The CRT is a measure of the tendency of people to avoid giving an immediate (but clearly wrong) answer (Frederick 2005). More recently, Markovits (2024) found that this extended to the scale of Actively Open-Minded Thinking (AOT), which is a measure of the tendency of people to consider alternative options when reasoning and has been found to correlate with deductive performance (Stanovich and West 1997).

There are two interesting aspects to these results. The first is the fact that the strategy diagnostic can successfully distinguish clear differences in a variety of different measures of cognitive ability. For example, using normalized scores on IQ, CRT, and AOT measures, Markovits (2024) found that Counterexample reasoners had a combined score (M = 0.438) that was much higher than Statistical reasoners (M = −0.099), with this difference being similar across all three measures. Second, this raises the question of whether the observed relationship between strategy use and various forms of reasoning and judgments tasks might simply be due to the fact that Counterexample reasoners are smarter, less prone to immediate wrong answers and more flexible in their judgments. There is, however, evidence that differences in performance related to strategy use are not solely a product of underlying differences in cognitive capacity. In the first direct examination of the potential role of differences in cognitive capacity on the effect of reasoning, (de Chantal et al. 2019) examined performance on syllogisms for which logical validity and conclusion belief are in conflict, leading to the belief-bias effect, which is one of the most extensively studies forms of heuristic bias (Evans et al. 1983). In their study, de Chantal et al. (2019) measured both performance on belief-biased inferences and working memory capacity. They found that Counterexample reasoners were significantly less prone to belief-bias effects, even when differences in working memory are factored out. In a recent more extensive study, Thompson and Markovits (2021) examined the respective contributions of strategy use, IQ, CRT, and numeracy on four different heuristic effects, belief-bias, base rate neglect, the conjunction fallacy and denominator neglect. They found that strategy use accounted for a significant proportion of the variance in belief-bias, base rate neglect and denominator neglect over and above the contribution of the other cognitive measures. Importantly, the latter two do not involve deductive reasoning, but involve judgment under uncertainty which gives additional evidence of the generality of the strategy use distinction.

Even stronger evidence for the unique contribution of strategy use is given by results examining the respective contributions of strategy use and cognitive capacity on performance under severe time constraint. These studies specifically examined the ability of participants to produce “valid” responses when given standard belief-biased syllogisms. There are two models that suggest that this ability should be strongly correlated with cognitive capacity. The first is the standard dual-process model that suggests that making valid deductions to such syllogisms (and not giving the heuristic belief-driven response) is generally acknowledged to require Type 2 conscious, working memory-dependent processes (De Neys 2006). Thus, reducing the time allotted for making inferences should decrease the rate at which the valid response is chosen (Evans and Curtis-Holmes 2005), and should make differences in working memory correspondingly more important. Another approach to fast reasoning considers that people might have access to a form of “logical” intuition which would allow generating the valid response without using processes that require cognitive capacity (De Neys 2012; De Neys and Pennycook 2019). Thus, use of such intuitive reasoning would allow fast logical reasoning that does not depend on working memory-dependent processes. However, there is evidence that the ability to make intuitive responses that are logical is concentrated among better reasoners (Raoelison et al. 2020), and thus, fast logical reasoning should be strongly associated with increased cognitive capacity.

In two studies, the ability to make fast, logical inferences was examined along with measures of strategy use and measures of cognitive capacity. The results obtained by Markovits et al. (2021) and Markovits (2023) clearly show that strategy use is a strong predictor of the ability to reason logically under severe time constraints, even when cognitive capacity is factored out. These results are particularly striking since they also show that, in contrast with studies examining reasoning under normal conditions, once strategy use is considered, other measures of cognitive capacity have little effect.

## 3. Strategy and Information Processing

These results clearly show that the relation between strategy use and performance on a variety of reasoning and judgment tasks cannot be explained solely by differences in cognitive capacity. They suggest that the impact of reasoning strategy is due to differences in the way that information is processed during reasoning. The general description of the underlying processes characterizing mental model-like and probabilistic forms of reasoning suggests some such differences. The first of these is attentional. The key to this lies with the idea that when people are reasoning, they will access not only the information that is directly given with the problem, but other forms of information that are related, but not explicitly presented. The most direct evidence for this is the existence of very clear content effects in simple conditional reasoning problems (Cummins 1995; Markovits and Vachon 1990; Thompson 1995). Results indicating that people’s inferences on what are formally identical forms of reasoning clearly suggest that when reasoning, information semantically associated with problem parameters is activated along with information that is directly presented with the inference (see Markovits 2014 for a review).

The description of the underlying processes that distinguish the two strategies suggests that the way that this extended representation is processed will vary according to strategy use. When given an inferential problem, Statistical reasoners attempt to determine the relative probability that a given conclusion or judgment is true, given the reasoners’ stored contextual knowledge. In other words, these reasoners will tend to consider a broad range of information when making an inference and will be strongly influenced by the associative relations between these elements. By contrast, Counterexample reasoners will focus on a smaller range of information in order to construct an internal model of the inference. The subset of information that is incorporated into the model will depend on what is considered to be particularly salient. In order to do this, it is necessary to attempt to inhibit activation of information not in this subset.

There are two predictions that can be made using this model. The first is that when faced with inferences for which there is a conflict between problem premises and associated information particularly conclusion believability, Counterexample reasoners will attend more to premises and less to conclusions. In (de Chantal et al. 2019), the authors used eye-tracking to examine focal attention with different forms of syllogisms, including belief-biased ones, and standard syllogisms where conclusions were belief neutral. They found that for the latter forms of deduction, Counterexample and Statistical reasoners showed the same patterns of attention, focusing to the same extent on either the premises or the conclusion. However, when conclusions were believable or unbelievable and this was in conflict with logical validity, Statistical reasoners focused more often on the conclusion than did Counterexample reasoners. In other words, when faced with a conflict between inferences that are directly suggested by problem premises and semantic information concerning the status of a putative conclusion, Statistical reasoners pay more attention to the former while Counterexample reasoners do the inverse.

A second prediction is related to the first. This is the idea that Statistical reasoners will be more affected by peripheral information when reasoning or making judgments. One example of this is belief-biased inferences, where the logical conclusion is that which can be generated by solely considering problem premises, while being influenced by the believability of a putative conclusion requires activating information peripheral to premises. This would suggest that Statistical reasoners would be more influenced by conclusion belief than Counterexample reasoners. Two studies have examined the relation between performance on belief-biased inferences and strategy use. Their results clearly show that Statistical reasoners are more influenced by conclusion belief than Counterexample reasoners (de Chantal et al. 2019; Markovits et al. 2017).

These studies examine classical reasoning tasks. The dual-strategy model also suggests that such attentional differences should be more domain general and generalize to forms of reasoning that do not involve judgments of validity. More specifically, the strategy distinction has been used to examine the relative impact of biases when reasoning about the social world. Such reasoning is ubiquitous and generally requires processing a large quantity of information. In such situations, people tend to use heuristic short-cuts. Such heuristics are stored in memory and are often, although not always, stereotypical in nature. This led to the hypothesis that Statistical reasoners would be more influenced by such social biases, which was indeed confirmed in a series of three studies examining three different forms of social biases (Gagnon-St-Pierre et al. 2020). Of particular interest in these studies was the effect of using an essentialism prime which has been found to increase levels of sexism. The results showed that this was true for Statistical reasoners, but not for Counterexample reasoners. This reinforces the basic idea that the former are more sensitive to activation of distant information, and thus more susceptible to the effects of explicitly activating biases.

Other studies have examined the impact of strategy differences in forms of information processing which have shown gender differences. The basic idea underlying these studies is the idea that well-documented gender differences should be the result of the use of some form of intuitive schema. Since such schemas would be preferentially activated by Statistical reasoners, this led to the hypothesis that these gender differences should be more observable within Statistical than Counterexample reasoners. Two separate studies have examined gender differences within the context of strategy use. An initial study looked at the well-documented gender difference in the processing of negative emotions. Studies have shown that females process negative emotions faster than males. In a series of two studies, it was found that this difference was concentrated among Statistical reasoners, with gender differences being less strong among Counterexample reasoners (Gagnon-St-Pierre et al. 2020). A second set of studies examined gender differences in mental rotation, which have generally been found to have a clear gender difference, with males performing better than females (Voyer 2011). Once again, the gender difference was found to be modulated by strategy use.

These studies provide clear evidence for the basic idea that reasoners using a Statistical strategy are more sensitive to the effects of information that may not be central to a given form of reasoning or judgment, but that are associated semantically with the parameters of the problem, compared to Counterexample reasoners.

## 4. Strategy Use and Susceptibility to Disconfirming Information

Another dimension in the way that strategy use determines differences in the way that information is processed is the weight given to information that contradicts a potential conclusion or judgment. Mental model theories put a great deal of weight on such information, when it is integrated into the representation that is made of problem parameters. By contrast, a probabilistic evaluation considers this information in the context of the sum of existing information, which should reduce its relative impact. The prediction that Counterexample reasoners should be more affected by negative information than Statistical reasoners has been confirmed in studies examining very different forms of judgment. For example, a recent study has found that Counterexample reasoners are more prone to revise judgments of false news items that have been reinforced by repeated presentation than Statistical reasoners following a single disconfirmation (Gratton and Markovits 2021). A recent series of studies have examined strategy differences in the way that contingency information is translated into perceptions of causality. Contingency information can be divided into two broad categories, “positive” information that suggests the existence of a causal link between a given cause and a given effect, and “negative” information that contradicts this link. Counterexample reasoners have been found to give more weight to the latter than Statistical reasoners (Béghin et al. 2021; Béghin and Markovits 2022, 2023). Finally, the same basic difference has been found when the way that information that is inconsistent with a possible conclusion in conditional inferences is processed, with Counterexample reasoners being more conservative, thus accepting inferences less frequently on the basis of the same information than Statistical reasoners (Brisson and Markovits 2020).

## 5. Strategy and Speed of Processing

One further distinction that is important concerns the relative speed at which the two strategies function. Theoretically, statistical reasoners who use more intuitive access to information required to estimate probabilities should make decisions more rapidly than counterexample reasoners who must make a more explicit selection of information and must integrate this into some kind of internal model. There is clear evidence that supports this distinction. Markovits et al. (2013) analyzed the time taken to respond to the diagnostic questionnaire when participants were given as much time as they wanted. This showed that responding with a Counterexample strategy was significantly faster than responding with a Statistical strategy. This same pattern is found when performance on other reasoning tasks are examined in the light of the strategy distinction. Performance on belief-biased inferences has shown that Statistical reasoners are generally faster than Counterexample reasoners, when given these kinds of inference (Markovits et al. 2017). Although more indirect, previously cited results showing that when giving belief-biased inferences with a strong time constraint, strategy use is a much better predictor of performance than cognitive capacity (Markovits et al. 2021), support this idea. These results are consistent with the idea that people using a Statistical strategy are generally faster and use a mode of processing that is less dependent on cognitive resources. A recent study has provided a clearer picture of the way that differential time constraints impact the way that strategy affects reasoning (Markovits 2024). In this study, participants were presented with an initial set of belief-biased problems for which they were given as much time as they wanted. They then received a second set of equivalent, although not identical, problems with a very severe time constraint (4 s). The results showed an overall decrease in the level of “correct” responses in the time constrained condition, as would be expected under most models. However, this was only found among Counterexample reasoners, with Statistical reasoners showing very little change in performance. This provides clear evidence that Counterexample reasoners use processes that are more cognitively demanding than Statistical reasoners, while the latter are using less demanding processes that are more “intuitive”, and generally require less time.

## 6. How Invariant Are Strategies?

The studies that have examined how strategy use is correlated with reasoning and judgment across a variety of different domains have operated under the basic premise that people have a preferred way of processing information. The results of these studies are certainly consistent with this idea and have clearly show that, at least in the very short term, the strategy distinction is correlated with the way that information is processed in a variety of forms of judgment and reasoning. The relationship between various measures of cognitive capacity and strategy use are also consistent with Verschueren’s hypothesis that a Counterexample strategy would be preferentially adopted by people with greater cognitive capacity, for the simple reason that this form of information processing is more cognitively demanding. However, another question then is the malleability of strategy use. Two sets of results provide some answers to this question. First, Markovits et al. (2013) presented participants with the strategy diagnostic both with unlimited time and under time constraint. They also asked participants to give a metacognitive evaluation of their confidence in their choices. The main results showed that when under an initial time constraint, people produced a much higher proportion of Statistical strategies compared to participants who performed the diagnostic with unlimited time. When making the diagnostic under unlimited time conditions, and then with a time constraint, Counterexample reasoners remained relatively stable, while a certain proportion of Statistical reasoners generated a Counterexample strategy. In addition, in a second study, the extent to which people modified their strategy when first given a time constraint followed by a second diagnostic with no constraint was related to their metacognitive evaluations of their time constrained strategy. When participants had higher levels of confidence in the strategy that they used under time constraint, they were more likely to maintain this when given unlimited time. These results show firstly that the Statistical strategy appears to be a default method of processing under steep constraint, which is consistent with the idea that this is a lower-cost form of information processing. It should be noted that this is also consistent with developmental results that show that very young children are able to make simple probabilistic judgments earlier than simple judgments of validity (Markovits and Thompson 2008). The other aspect of these results is the relation between metacognitive evaluations and strategy change. This suggests that, at some level, strategy use is under some degree of control, which in turn suggests that it might be possible to induce people to modify their preferred strategy.

This hypothesis was examined in a recent study. Markovits and Thompson (2023) first evaluated initial strategy use. Following this, they presented participants with one of two training procedures that were designed to induce the use of either a counterexample or a probabilistic mode of evaluating possible conclusions. Immediately after the training procedure, participants were given two sets of updating problems, taken from (Markovits et al. 2015), that were designed to indicate to what extent participants were able to successfully follow the training procedure. After this, in Study 1 participants were given a set of belief-biased problems, while in Study 2, they received a set of base rate problems. The results showed two clear effects. First, people’s ability to successfully follow the instructions given in the training procedures was strongly related to their initial strategy use. Specifically, Counterexample reasoners found it easier to follow counterexample instructions, while Statistical reasoners found it easier to follow probabilistic instructions. Independently of the transfer effect, this provides additional evidence that the strategy diagnostic taps into a fundamental difference in the way that people process information. Second, both studies showed that Statistical reasoners who were able to successfully follow instructions performed differently on the subsequent problems, while Counterexample reasoners showed relatively little change. Although the direction of change varied between the belief-biased and the base rate problems, the key results were the fact that a short-term instructional procedure induced clear changes in the way that Statistical reasoners performed, with the effects on Counterexample reasoners being less marked.

What these results show is that strategy use, though relatively stable in the short term under normal conditions, is not invariant. People who spontaneously use a Statistical strategy can be encouraged to use a Counterexample strategy to some extent with a fairly simple intervention, while those spontaneously using a Counterexample strategy remain with this. The Statistical strategy is nonetheless a default strategy, such that when people are asked to make immediate judgments under extreme constraint, they tend to fall back on the Statistical strategy. Interestingly, this is much less the case when people are initially given problems under normal conditions, after which time constraints have much less of an effect.

## 7. Extended Analysis of Strategy Diagnostic

The studies that have been referred to specifically examine the distinction between Counterexample and Statistical strategies. However, these studies have generally found that only about 2/3 of participants can be classified in one of these two categories. Recent studies have begun to examine response patterns that were not previously considered. These have indicated the existence of two subgroups, one of which is composed of people who tend to use a Counterexample strategy, but sometimes default to a Statistical strategy, which we refer to as the Intermediate category (note that the explicit criterion for the Intermediate category is rejecting four of the five 90% inferences in the strategy diagnostic). The Other category, which is everyone not in any of the three defined category, appears to consist of participants who have difficulty in understanding the basic parameters of the problems used in the diagnostic questionnaire. This latter category is consistent with Stanovich’s idea that some people are bad reasoners since they lack appropriate mindware (Stanovich 2018).

There are two kinds of results that support these additional categories. When the extended strategy categories are correlated with measures of cognitive capacity, Intermediate reasoners have levels of cognitive capacity that lie between those of Counterexample and Statistical reasoners, while the Other category of participants have much lower levels than the latter. This has been found for IQ and CRT measures (Thompson and Markovits 2021) and for IQ, CRT, and AOT (Markovits 2024). The second concerns the way that the different categories respond to two different kinds of manipulation. When given instructions to reason using either counterexamples or probabilities (Markovits and Thompson 2023), the ability to successfully follow counterexample instructions shows a linear decrease between Counterexample, Intermediate, Statistical, and Other reasoners. In addition, when participants are given an initial set of belief-biased problems under normal conditions followed by a second set under time constraint (Markovits 2024), Intermediate reasoners show clear effects of time constraint, albeit one that is less than that observed in Counterexample reasoners, while both Statistical and Other reasoners show no effect of time constraint.

The existence of the Intermediate category is particularly interesting, since it suggests, in line with previously discussed results that there is a certain, albeit limited, form of flexibility in strategy use. This appears to be a function of the level of cognitive ability, with reasoners whose level of ability is not high enough to be comfortable relying solely on a Counterexample strategy occasionally defaulting to a Statistical strategy.

## 8. Developmental Considerations

The dual-strategy model has been developed to describe adult reasoning. Extending this model to younger ages is certainly an important direction for future research. Nonetheless, there are some results from earlier studies that suggest that the strategy distinction might indeed be relevant in children. The first is a study by Markovits and Thompson (2008) which examined probabilistic and counterexample reasoning in 6- to 9-year-old children. The results showed that while both forms of reasoning can be seen in 6-year-olds, explicitly counterexample reasoning undergoes a clear increase over this period, while probabilistic reasoning remains relatively constant. Markovits et al. (2019) examined developmental patterns of both forms of reasoning among 8- and 10-year-olds under time constraint. Their results show that time constraint results in parallel decreases in both forms of reasoning. These results suggest that even young children have access to both forms of reasoning, and that they undergo clear developmental increases. They also suggest that while probabilistic reasoning remains fairly intuitive at all ages, development of counterexample reasoning might correlate more closely with more advanced reasoning skills at later ages, although this remains speculative.

## 9. Conclusions

Based on an initial proposal by (Verschueren et al. 2005b), the dual-strategy model has identified two different modes of reasoning or making various kinds of judgments that mirror the distinction between mental model and probabilistic theories of reasoning. Counterexample reasoners construct limited representations of problem parameters which are particularly sensitive to potential counterexamples to possible conclusions. Statistical reasoners generate likelihood estimates that incorporate a wider range of information. Counterexample reasoners have greater levels of cognitive capacity and use procedures that are more working memory intensive and require relatively more time compared to Statistical reasoners who use a more intuitive and faster form of processing.

Existing results clearly show that strategy use is a stable short term individual difference which predicts differences both in classical forms of reasoning and also in wider forms of judgment. The two strategies are mutually exclusive, although there is some evidence that people sometimes alternate between them in successive judgments. Strategy use defines a clear difference in the way that information is processed, one that appears to have broader effects above that observed on the standard reasoning tasks that were used to define the dual-strategy model.

There are some general implications of this model. First, there is a strong relationship between strategy use and several measures of cognitive capacity, with Counterexample reasoners having higher levels of several measures related to both capacity and individual differences in reasoning (such as Actively Open-Minded Thinking). Previous results have shown that while strategy use is correlated with measures of capacity, it accounts for significant variance over and above the effects of cognitive capacity in a variety of forms of reasoning and, as is the case for very fast reasoning, can be a much better predictor than capacity. Thus, differences related to cognitive capacity might partially or totally reflect underlying differences in strategy use. The extent to which these differences are due to real differences in cognitive capacity or to processing differences that are correlated with capacity remains an open question.

Another important component of this model is the observation that, while strategy use is relatively stable, it is somewhat variable. This is particularly the case when people are time constrained when making judgments, and presumably when subject to other forms of constraint, where there is a tendency to use a Statistical strategy which appears to be a low-cost default mode of processing. This implies that performance differences observed when conditions change might be the result of changing strategies as much as limitations in the procedures that are normally used.

In sum, these results have identified an important individual difference in the way that people reason and make judgments in a variety of domains. Although the underlying models propose different kinds of algorithms that can be used to reason, the key observation is that these two strategies imply important differences in the way that information is processed.

## Data Availability

Not applicable.

## Appendix A

**Diagnostic questionnaire**

The full questionnaire is presented in the following passages. This presents 10 problems involving inferences of the form “P implies Q, Q is true. Is “P is true” a valid conclusion”. For each of these problems, the results of 1000 observations are given; these show the number of times that P and Q were observed together, and the number of times that P and not-Q were observed together. For five of these problems, the percentage of times that P and Q were observed together was about 50%, while for the other five, this percentage was about 90%. Three filler problems presented inferences of the form “P implies Q. P is true. Is “Q is true” a valid conclusion”.

Coding scheme.

R50 is the number of times (of a total of 5) that the conclusion was not considered to be valid for the 50% problems.

R90 is the number of times (of a total of 5) that the conclusion was not considered to be valid for the 90% problems.

The algorithm for classifying reasoners is the following:

R50 > R90 + 1 then **Statistical**

R90 + R50 = 10 then **Counterexample**

R90 = 4 then **Intermediate**

Otherwise **Other**

**General Instructions**

Imagine that a team of scientists are on an expedition to a new planet that has recently been discovered, **Planet Kronus**. In the following, we will ask you to perform some special exercises. You will be asked to respond to questions about specific things that are only found on Kronus, and which do not exist on the Earth. You will receive information in the form of rules, which you must consider to be true. It is very important that when you respond to the questions that you must suppose that the presented rule is always true. Following each rule, you will see an observation and a conclusion. You must indicate whether the conclusion follows logically or not from the information that is presented.

Problem 1:

A team of meteorologists watching the local climate of the planet Kronus noted an interesting phenomenon. They noted that on Kronus:

**If it thardonnes, then the ground will become sticky.**

On the last 1000 times it thardonned, meteorologists made the following observations:

**1000 times it thardonned and the ground became sticky.**

**0 times it has not thardonned and the ground became sticky.**

**From this information, John reasoned as follows:**

If it thardonnes, then the ground will become sticky.

Observation: It thardonnes.

Conclusion: The ground will become sticky.

**Indicate whether you believe this conclusion can be drawn logically or not from the information provided.**

Yes No

Problem 2:

While exploring a cave on Kronus, geologists discovered a variety of a very special stone, a trolyte. Following a series of observations, they claim that on Kronus:

**If a trolyte is heated, then it will release philoben gas.**

Of the last 1000 times they observed trolytes, geologists made the following observations:

**900 trolytes were heated and released philoben gas.**

**100 trolytes were not heated and released philoben gas.**

**From this information, John reasoned as follows:**

Geologists claim that: If a trolyte is heated, then it will release philoben gas.

Observation: A trolyte released philoben gas.

Conclusion: The trolyte was heated.

**Indicate whether you believe this conclusion can be drawn logically or not from the information provided.**

Yes No

Problem 3:

By studying the unique wildlife of Kronus, biologists have made a discovery about the birds of Kronus. They state that:

**If a bird has water in its beak, then the color of its plumage will change.**

Of the 1000 birds they examined recently, the scientists made the following observations:

**500 birds had water in their beak and the color of their plumage changed.**

**500 birds have not had water in their beak and the color of their plumage changed.**

**From this information, John reasoned as follows:**

Biologists stated that: If a bird has water in its beak, then the color of its plumage will change.

Observation: The color of the plumage of a bird has changed.

Conclusion: The bird has water in its beak.

**Indicate whether you believe this conclusion can be drawn logically or not from the information provided.**

Yes No

Problem 4:

A group of botanists studying the flora has discovered a unique property of plants growing on Kronus. According to the botanists, on Kronus:

**If X45 fertilizer is given to a plant, then the plant will become phosphorescent.**

Of the last 1000 times they observed the plants, the scientists made the following observations:

**1000 plants were given X45 fertilizer and became phosphorescent.**

**0 plants were not given X45 fertilizer and became phosphorescent.**

**From this information, John reasoned as follows:**

The botanists claim that: If X45 fertilizer is given to a plant, then the plant will become phosphorescent.

Observation: A plant is given X45 fertilizer.

Conclusion: The plant will become phosphorescent.

**Indicate whether you believe this conclusion can be drawn logically or not from the information provided.**

Yes No

Problem 5:

A team of chemists mixed different substances unique to the planet Kronus. Following a series of observations, they affirm that on Kronus:

**If fannar is mixed with water, then it will become yellow.**

On the last 1000 observations made, the chemists noted that:

**910 times fannar was mixed with water and became yellow.**

**90 times fannar was not mixed with water and became yellow.**

**From this information, John reasoned as follows:**

The chemists affirm that: If fannar is mixed with water, then it will become yellow.

Observation: Some fannar has become yellow.

Conclusion: The fannar has been mixed with water.

**Indicate whether you believe this conclusion can be drawn logically or not from the information provided.**

Yes No

Problem 6:

A team of meteorologists observing the local climate of the planet Kronus noted an interesting phenomenon. They noted that on Kronus:

**If the sun shines, then the ground will become green.**

On the last 1000 times when they observed the ground, meteorologists made the following observations:

**505 times the sun was shining and the ground became green.**

**495 times the sun was not shining and the ground became green.**

**From this information, John reasoned as follows:**

The meteorologists affirmed that: If the sun shines, then the ground will become green.

Observation: The ground became green.

Conclusion: The sun was shining.

**Indicate whether you believe this conclusion can be drawn logically or not from the information provided.**

Yes No

Problem 7:

Chemists working on Kronus found that the water was very special. Following a series of observations, they affirmed that on Kronus:

**If water is boiled, then the water will become red.**

On the last 1000 experiments they made, chemists made the following observations:

**1000 times, water was boiled and it became red.**

**0 times, water was not boiled and it became red.**

**From this information, John reasoned as follows:**

Chemists affirmed that: If water is boiled, then the water will become red.

Observation: Water is boiled.

Conclusion: The water will become red.

**Indicate whether you believe this conclusion can be drawn logically or not from the information provided.**

Yes No

Problem 8:

While exploring a cave on Kronus, geologists discovered a particular property of rocks. Following a series of observations, they claim that on Kronus:

**If a rock is made wet, then it will change color.**

Over the last 1000 times they observed rocks, geologists made the following observations:

**920 rocks were made wet and changed color.**

**80 rocks were not made wet and changed color.**

**From this information, John reasoned as follows:**

Geologists claim that: If a rock is made wet, then it will change color.

Observation: A rock changes color.

Conclusion: The rock was made wet.

**Indicate whether you believe this conclusion can be drawn logically or not from the information provided.**

Yes No

Problem 9:

When studying the unique wildlife of Kronus, biologists made a discovery about cats on Kronus. They claim that:

**If you feed a cat, then its eyes turn red.**

Of the 1000 cats they examined, the scientists made the following observations:

**510 cats were fed and their eyes turned red.**

**490 cats were not fed and their eyes turned red.**

**From this information, John reasoned as follows:**

Biologists claim that: If you feed a cat, then its eyes turn red.

Observation: A cat has red eyes.

Conclusion: The cat was fed.

**Indicate whether you believe this conclusion can be drawn logically or not from the information provided.**

Yes No

Problem 10:

A group of botanists studying the flora discovered another unique property of plants growing on Kronus. According to the botanists, on Kronus:

**If a plant is watered, then the plant will become orange.**

Of the last 1000 times they observed plants, the scientists made the following observations:

**905 plants were watered and became orange.**

**95 plants were not watered and became orange.**

**From this information, John reasoned as follows:**

Botanists claim that: If a plant is watered, then the plant will become orange.

Observation: A plant became orange.

Conclusion: The plant was watered.

**Indicate whether you believe this conclusion can be drawn logically or not from the information provided.**

Yes No

Problem 11:

A team of biologists on Kronus has discovered a very special animal, a kikina. Following a series of observations on this animal, they claim that on Kronus:

**If a kikina eats meat, then it will shrink.**

Of the last 1000 experiments they made, the biologists made the following observations:

**520 kikinas ate meat and shrunk.**

**480 kikinas did not eat meat and shrunk.**

**From this information, John reasoned as follows:**

Biologists claim that: If a kikina eats meat, then it will shrink.

Observation: A kikina has shrunk.

Conclusion: The kikina ate meat.

**Indicate whether you believe this conclusion can be drawn logically or not from the information provided.**

Yes No

Problem 12:

A group of botanists studying the different trees on Kronus discovered that they have a unique property. According to the botanists, on Kronus:

**If you burn a tree, then purple smoke will be produced.**

Of the last 1000 observations, the scientists made the following observations:

**915 times trees were burned and purple smoke was produced.**

**85 times trees were not burned and purple smoke was produced.**

**From this information, John reasoned as follows:**

The botanists claim that: If you burn a tree, then purple smoke will be produced.

Observation: Purple smoke was produced.

Conclusion: A tree was burned.

**Indicate whether you believe this conclusion can be drawn logically or not from the information provided.**

Yes No

Problem 13:

A team of biologists has discovered on Kronus an animal having unique properties, the tritana. Following a series of observations, they claim that on Kronus:

**If a tritana is sprayed with alcohol, then it will emit a high-pitched sound.**

Of the last 1000 experiments they made, the geologists made the following observations:

**515 tritana were sprayed with alcohol and emitted a high-pitched sound.**

**485 tritana were not sprayed with alcohol and emitted a high-pitched sound.**

**From this information, John reasoned as follows:**

Biologists claim that: If tritana is sprayed with alcohol, then it will emit a high-pitched sound.

Observation: A tritana emits a high-pitched sound.

Conclusion: The tritana was sprayed with alcohol.

**Indicate whether you believe this conclusion can be drawn logically or not from the information provided.**

Yes No

## References

[B1-jintelligence-12-00025] Béghin Gaetan, Markovits Henry (2022). Reasoning strategies and prior knowledge effects in contingency learning. Memory & Cognition.

[B2-jintelligence-12-00025] Béghin Gaetan, Markovits Henry (2023). Interpretation of ambiguous trials along with reasoning strategy is related to causal judgements in zero-contingency learning. Quarterly Journal of Experimental Psychology.

[B3-jintelligence-12-00025] Béghin Gaetan, Gagnon-St-Pierre Émilie, Markovits Henry (2021). A dual strategy account of individual differences in information processing in contingency judgments. Journal of Cognitive Psychology.

[B4-jintelligence-12-00025] Brisson Janie, Markovits Henry (2020). Reasoning strategies and semantic memory effects in deductive reasoning. Memory & Cognition.

[B5-jintelligence-12-00025] Cummins Denise (1995). Naive theories and causal deduction. Memory & Cognition.

[B6-jintelligence-12-00025] de Chantal Pier-Luc, Newman Ian, Thompson Valerie A., Markovits Henry (2019). Who resists belief-biased inferences? The role of individual differences in reasoning strategies, working memory, and attentional focus. Memory & Cognition.

[B7-jintelligence-12-00025] De Neys Wim (2006). Dual Processing in Reasoning: Two Systems but One Reasoner. Psychological Science.

[B8-jintelligence-12-00025] De Neys Wim (2012). Bias and conflict: A case for logical intuitions. Perspectives on Psychological Science.

[B9-jintelligence-12-00025] De Neys Wim, Pennycook Gordon (2019). Logic, fast and slow: Advances in dual-process theorizing. Current Directions in Psychological Science.

[B10-jintelligence-12-00025] Evans Jonathan St. B. (2007). Hypothetical Thinking: Dual Processes in Reasoning and Judgement.

[B11-jintelligence-12-00025] Evans Jonathan St. B., Curtis-Holmes Jody (2005). Rapid responding increases belief bias: Evidence for the dual-process theory of reasoning. Thinking & Reasoning.

[B12-jintelligence-12-00025] Evans Jonathan St. B., Stanovich Keith E. (2013). Dual-process theories of higher cognition advancing the debate. Perspectives on Psychological Science.

[B13-jintelligence-12-00025] Evans Jonathan St. B., Over David E., Handley Simon J. (2003). Conditionals and conditional probability. Journal of Experimental Psychology: Learning, Memory, and Cognition.

[B14-jintelligence-12-00025] Evans Jonathan St. B., Barston Julie L., Pollard Paul (1983). On the conflict between logic and belief in syllogistic reasoning. Memory & Cognition.

[B15-jintelligence-12-00025] Frederick Shane (2005). Cognitive reflection and decision making. Journal of Economic Perspectives.

[B16-jintelligence-12-00025] Gagnon-St-Pierre Émilie, Doucerain Marina M., Markovits Henry (2020). Reasoning strategies explain individual differences in social reasoning. Journal of Experimental Psychology: General.

[B17-jintelligence-12-00025] Geiger Sonja, Oberauer Klaus (2007). Reasoning with conditionals: Does every counterexample count? its frequency that counts. Memory & Cognition.

[B18-jintelligence-12-00025] Gratton Cloé, Markovits Henry (2021). Reasoning strategies determine the effect of disconfirmation on belief in false claims. Memory & Cognition.

[B19-jintelligence-12-00025] Hinterecker Thomas, Knauff Markus, Johnson-Laird Philip N. (2016). Modality, probability, and mental models. Journal of Experimental Psychology: Learning, Memory, and Cognition.

[B20-jintelligence-12-00025] Johnson-Laird Philip. N. (2001). Mental models and deduction. Trends in Cognitive Sciences.

[B21-jintelligence-12-00025] Johnson-Laird Philip N. (2012). Inference with mental models. The Oxford Handbook of Thinking and Reasoning.

[B22-jintelligence-12-00025] Markovits Henry, Feeney Aidan, Thompson Valerie A. (2014). Conditional reasoning and semantic memory retrieval. Reasoning and Memory.

[B23-jintelligence-12-00025] Markovits Henry (2023). Fast logic and belief-bias: It’s not how smart you are, but how you think. Journal of Cognitive Psychology.

[B24-jintelligence-12-00025] Markovits Henry (2024). Reasoning strategy moderates the transition between slow and fast reasoning. Journal of Cognitive Psychology.

[B31-jintelligence-12-00025] Markovits Henry, Vachon Robert (1990). Conditional reasoning, representation, and level of abstraction. Developmental Psychology.

[B33-jintelligence-12-00025] Markovits Henry, Thompson Valerie A. (2008). Different developmental patterns of simple deductive and probabilistic inferential reasoning. Memory and Cognition.

[B34-jintelligence-12-00025] Markovits Henry, Thompson Valerie A. (2023). Can we change how people reason? Effects of instructions to reason differently and reasoning strategy. Journal of Experimental Psychology: Learning, Memory and Cognition.

[B25-jintelligence-12-00025] Markovits Henry, Forgues Hugues Lortie, Brunet Marie-Laurence (2012). More evidence for a dual-process model of conditional reasoning. Memory and Cognition.

[B26-jintelligence-12-00025] Markovits Henry, Brisson Janie, de Chantal Pier-Luc (2015). Deductive updating is not Bayesian. Journal of Experimental Psychology: Learning, Memory, and Cognition.

[B27-jintelligence-12-00025] Markovits Henry, Brisson Janie, de Chantal Pier-Luc (2016). Logical Reasoning Versus Information Processing in the Dual-Strategy Model of Reasoning. Journal of Experimental Psychology. Learning, Memory, and Cognition.

[B28-jintelligence-12-00025] Markovits Henry, Brisson Janie, de Chantal Pier-Luc, Thompson Valerie A. (2017). Interactions between inferential strategies and belief bias. Memory & Cognition.

[B29-jintelligence-12-00025] Markovits Henry, Brunet Marie-Laurence, Thompson Valerie A., Brisson Janie (2013). Direct evidence for a dual process model of deductive inference. Journal of Experimental Psychology: Learning, Memory, and Cognition.

[B30-jintelligence-12-00025] Markovits Henry, de Chantal Pier-Luc, Brisson Janie, Gagnon-St-Pierre Emilie (2019). The development of fast and slow inferential responding: Evidence for a parallel development of rule-based and belief-based intuitions. Memory and Cognition.

[B32-jintelligence-12-00025] Markovits Henry, de Chantal Pier-Luc, Brisson Janie, Dubé Éloise, Thompson Valerie A., Newman Ian (2021). Reasoning strategies predict use of very fast logical reasoning. Memory & Cognition.

[B35-jintelligence-12-00025] Oaksford Mike, Chater Nick (2009). Précis of Bayesian rationality: The probabilistic approach to human reasoning. Behavioral and Brain Sciences.

[B36-jintelligence-12-00025] Oaksford Mike, Chater Nick, Larkin Joanne (2000). Probabilities and polarity biases in conditional inference. Journal of Experimental Psychology: Learning, Memory, and Cognition.

[B37-jintelligence-12-00025] Raoelison Matthieu, Thompson Valerie A., De Neys Wim (2020). The smart intuitor: Cognitive capacity predicts intuitive rather than deliberate thinking. Cognition.

[B38-jintelligence-12-00025] Sloman Steven A. (1996). The empirical case for two systems of reasoning. Psychological Bulletin.

[B39-jintelligence-12-00025] Stanovich Keith E. (2018). Miserliness in human cognition: The interaction of detection, override and mindware. Thinking & Reasoning.

[B40-jintelligence-12-00025] Stanovich Keith E., West Richard F. (1997). Reasoning independently of prior belief and individual differences in actively open-minded thinking. Journal of Educational Psychology.

[B41-jintelligence-12-00025] Stanovich Keith E., West Richard F. (2000). Individual differences in reasoning: Implications for the rationality debate?. Behavioral and Brain Sciences.

[B42-jintelligence-12-00025] Thompson Valerie A. (1995). Conditional reasoning: The necessary and sufficient conditions. Canadian Journal of Experimental Psychology/Revue Canadienne de Psychologie Expérimentale.

[B43-jintelligence-12-00025] Thompson Valerie A., Markovits Henry (2021). Reasoning strategy vs cognitive capacity as predictors of individual differences in reasoning performance. Cognition.

[B44-jintelligence-12-00025] Verschueren Niki, Schaeken Walter, d’Ydewalle Géry (2005a). A dual-process specification of causal conditional reasoning. Thinking & Reasoning.

[B45-jintelligence-12-00025] Verschueren Niki, Schaeken Walter, d’Ydewalle Géry (2005b). Everyday conditional reasoning: A working memory-dependent tradeoff between counterexample and likelihood use. Memory & Cognition.

[B46-jintelligence-12-00025] Voyer Daniel (2011). Time limits and gender differences on paper-and-pencil tests of mental rotation: A meta-analysis. Psychonomic Bulletin & Review.

